# MicroRNA Expression Profile in the Patient's Plasma Exosomes of Alcohol‐Induced Osteonecrosis of Femoral Head: Potential Vascular Regulation Mechanism

**DOI:** 10.1111/jcmm.70382

**Published:** 2025-02-24

**Authors:** Wei Chen, Cheng Li, Zhe Yi, Gaojie Luo, Peiyao Zhang, Panfeng Wu, Fang Yu, Liming Qing, Juyu Tang

**Affiliations:** ^1^ Department of Hand and Microsurgery, Xiangya Hospital Central South University Changsha Hunan China; ^2^ Department of Hand Surgery, Beijing Jishuitan Hospital Capital Medical University Beijing China

**Keywords:** alcohol, exosome, hepatocyte, HIF‐1α, high‐throughput sequencing, miR‐155‐5p, osteonecrosis of the femoral head, vascular endothelial cells

## Abstract

The purpose of this study is to reveal the expression profile of microRNA (miRNA) in the plasma exosomes of patients with alcohol‐induced osteonecrosis of the femoral head (AIONFH), and to further explore the potential effect of alcohol‐stimulated hepatocyte exosomes on vascular endothelial cells (VECs). The total RNA of plasma exosomes of normal people (NC‐Exos) and patients with AIONFH (AI‐Exos) were extracted, followed by Illumina high‐throughput sequencing. GO and KEGG analyses were performed. MiR‐155‐5p in alcohol‐stimulated hepatocyte exosomes (AML‐Exos) was detected by RT‐PCR. The effects of AI‐Exos/AML‐Exos on the migration, proliferation, and apoptosis of VECs were detected by wound healing assay, CCK‐8 assay, and flow cytometry, respectively. The expression of HIF‐1α in VECs, a target gene of miR‐155‐5p, was detected by Western Blot and qRT‐PCR. High‐throughput sequencing showed that 82 miRNAs were differentially expressed in AI‐Exos. RT‐PCR results indicated that miR‐155‐5p, which targets to regulate HIF‐1α as confirm via dual‐luciferase assay, was the most significantly up‐regulated in AI‐Exos. Further studies demonstrated that miR‐155‐5p was specifically up‐regulated in AML‐Exos. Both AI‐Exos and AML‐Exos inhibited the proliferation and migration of VECs, promoted their apoptosis, and down‐regulated the expression of HIF‐1α and VEGFA. This study is the first to reveal the miRNAs expression profile in plasma exosomes of AIONFH patients and suggest a potential mechanism by which exosomes released from alcohol‐stimulated hepatocytes may regulate VECs in AIONFH.

## 1 Introduction

Osteonecrosis of the femoral head (ONFH) is a common and refractory diseases in orthopaedics, typically affecting young adults aged 20–40. Without active and effective treatment, most patients follow a progression of “necrosis‐collapse‐hip osteoarthritis‐disability.” The causes of ONFH can be categorised into traumatic and non‐traumatic. Among non‐traumatic ONFH, alcohol‐induced ONFH (AIONFH) accounts for 20%–45% of incidents [1]. Due to the rapid progression and high disability rate, AIONFH significantly impacts patents' quality of life and imposes substantial economic burdens on families and society. Several possible mechanisms of ONFH have been proposed, including vascular damage, mechanical stresses, increased intraosseous pressure, adipocyte dysfunction, defects in apoptosis and coagulation dysfunction [2]. However, most research has concentrated on steroid‐induced ONFH (SONFH), leaving uncertainties regarding whether the pathogenesis of AIONFH, particularly concerning alcohol's impact on the liver. Therefore, exploring the specific mechanism and developing effective prevention and treatment strategies for AIONFH has become a key research focus.

In recent years, with the advancement of high‐throughput sequencing technology, transcriptome has been widely used in the study of various diseases. Increasing attention has been given to the role of non‐coding RNA (ncRNA) such as miRNA, circRNA and lncRNA in diagnosis, staging, treatment, and prognosis of ONFH [3, 4]. MiRNAs are endogenous ncRNAs of approximately 22 nucleotides in length that regulates the gene expression and function at the post‐transcriptional level by forming silencing complex, degrading the target gene, or preventing translation [5]. Genome‐wide miRNA spectrum analysis has identified numerous differentially expressed miRNAs in serum and BMSCs of ONFH patients, suggesting their potential as diagnostic markers [6, 7, 8, 9, 10]. High‐dose alcohol impair angiogenesis by regulating the expression of angiogenesis‐related genes such as vascular endothelial growth factor (VEGF) and hypoxia‐inducible factor (HIF), potentially causing more severe vascular injury in the osteonecrosis area compared to SONFH [11]. Further studies have indicated that miRNA contribute the occurrence and development of AIONFH by regulating adipogenesis, osteogenesis, and angiogenesis [12, 13]. These findings underscore the critical role of miRNAs in understanding and potentially treating AIONFH.

Exosomes are rich in proteins, lipids, and nucleic acids, and they transmit crucial biological information between cells and organs. Cells will release nano‐sized vesicles, such as extracellular vesicles or exosomes, in response to stress or injury, which participate in the repair of injured tissue. Various reactive injury cytokines produced by alcohol can be packaged into exosomes and then migrate to adjacent cells or distant tissues [14]. As the primary metabolic organ of alcohol, the liver secretes exosomes into the blood in a dose‐dependent manner, ultimately affecting the liver and other organs [15, 16, 17, 18]. In addition, high‐dose alcohol consumption can inhibit angiogenesis by down‐regulating the expression of VEGFA and VEGFR2 [19]. Furthermore, genetic variations of alcohol dehydrogenase (ADH) and aldehyde dehydrogenase (ALDH), common in peripheral blood and organs of the East Asian population, contribute to AIONFH [20, 21]. These results suggest that the exosomes secreted by alcohol‐stimulated hepatocytes may affect the biological behaviour of vascular endothelial cells (VECs) and thus participate in the process of AIONFH. The primary role in this process is undoubtedly played by the biological information carried by the exosomes, such as miRNAs.

In contrast to previous studies, which have primarily focused on steroid‐induced ONFH, this study uniquely investigates the expression profile and potential mechanisms of miRNAs in peripheral plasma exosomes (AI‐Exos) of AIONFH patients using high‐throughput sequencing. This research addresses a significant gap by specifically examining the role of alcohol‐induced exosomal miRNAs in the pathogenesis of AIONFH. The key focuses of this study are twofold: firstly, to screen for differentially expressed miRNAs in AI‐Exos through high‐throughput sequencing, followed by constructing a target gene network map and predicting related pathways via bioinformatics analysis; secondly, to explore the impact of exosomes secreted by alcohol‐stimulated hepatocytes on vascular endothelial cells (VECs). These investigations lay a crucial foundation for uncovering the unique pathogenesis of AIONFH and potentially guiding the development of targeted prevention and treatment strategies.

## 2 Materials and Methods

### 2.1 Study Population

From 2021 to 2022, 10 AIONFH patients and 10 healthy people were recruited at Xiangya Hospital of Central South University, aged between 20 and 50 years old. This study was approved by the Xiangya Hospital Ethics Committee (no. 202004217), and informed consent was obtained from all participants.

Inclusion and exclusion criteria of the case group: (1) all the patients included in the case group met the alcohol intake standard for AIONFH diagnostic criteria proposed by the Association Research Circulation Osseous (ARCO) [22]; (2) patients at ARCO stage 2 or 3 diagnosed by clinical imaging examination (X‐ray, CT, MRI, etc.) were included; (3) the patients had not yet quit drinking at the time of study inclusion; (4) patients with non‐union or other types of hip joint diseases were excluded; (5) patients with haematological, mental and other serious medical diseases were excluded. (6) Patients < 10 years old and those who did not agree to participate in the study were excluded.

Inclusion and exclusion criteria of the healthy control group: (1) no hip joint disease and other orthopaedic diseases; (2) no blood system, mental system diseases, and other serious medical diseases; (3) no history of alcohol consumption, smoking, radiotherapy, hormone use and other factors; (4) no infections such as HIV, pancreatitis, osteomyelitis and other viruses; (5) no metabolic diseases such as diabetes, dyslipidemia, gout, pregnancy, etc. (6) No statistical difference compared to the case group.

### 2.2 Isolation and Characterisation of Exosomes

The blood was collected with EDTA tubes, and the blood cells were removed by centrifugation to get the plasma supernatant. The cell fragments and impurities in the plasma were removed by multiple rounds of centrifugation. For the extraction of human plasma exosomes, an equal volume of Blood PureExo Solution (BPS) precipitant (Umibio, UR52136) was added to the plasma, mixed thoroughly, and placed in the refrigerator at 4°C for 2 h, then centrifuged at 10,000 g at 4°C for 1 h to obtain the exosome precipitate. The exosomes were resuspended in PBS, purified using an Exosome Purification Filter (EPF) by centrifugation at 3000 g for 10 min, and stored for further use at −80°C. For the extraction of exosomes from AML‐12 mouse hepatocyte supernatant, the exosomes precipitant (SBI, EXOTCxxA‐1) was added at a 1/5 ratio to the supernatant, mixed, and placed at 4°C for 12 h. The mixture was then centrifuged at 1500 g for 30 min at 4°C to obtain exosomes. The resulting exosomes were resuspended in an appropriate amount of PBS, packed with 50–100 μL and stored at −80°C for follow‐up experiments. The total protein concentration was determined by the BCA protein quantitative kit (NCM Biotech, WB6501). The morphology of the exosomes was identified by TEM provided by the Electron microscope Room of the Pathology Research Center of Xiangya Hospital. The particle size of exosomes was analysed by NanoSight NS300 (Malvern Instruments). The expression of exosomes characteristic markers CD9, CD63, CD81, TSG101, and negative control HSP90B1 were detected by Western Blot.

### 2.3 Western Blot

Exosomes obtained by lysis buffer for exosome (Umibio, UR33101) were separated with 12% or 8% SDS‐PAGE and then transferred to the PVDF membrane. The blots were blocked by 5% bovine serum albumin (BSA) for 90 min at room temperature and incubated with primary antibodies at dilutions as instructed by the manufacturers at 4°C overnight. Then, the HRP‐conjugated secondary antibodies were added and incubated at room temperature for 1 h, and ECL detection reagents (BioSharp) were used to develop the blots. CD9, CD63, CD81, TSG101, and negative control HSP90B1 were detected as surface markers of exosomes. In VECs, the expression of HIF‐1α (Abcam, ab308433) and VEGFA (Abcam, ab229377) was detected, while β‐tubulin was used as the loading control. At least three parallel tests were carried out to confirm the final results and representative results were selected to show.

### 2.4 Extraction and Purification of RNA

RNAex (AccurateBiology, AG21102) was added to the exosomes at 1:5 proportion. Chloroform was used to extract RNA, and the appropriate amount of ethanol was added to the obtained supernatant after centrifugation. The total RNA of exosomes was purified according to the instructions of the RNA purification kit (Norgen, Cat.35300). The purity and concentration of total RNA were determined by a bioanalyzer (Agilent, 2100 Bioanalyzer). The RNA extraction of human umbilical vein epithelial cells (HUVECs) was performed according to the RNAex instruction (AccurateBiology, AG21102). The extraction process of exosomes and RNA is shown in Figure S1.

### 2.5 Real‐Time RT‐PCR

For the total RNA extracted from the exosomes, the reagent was added according to the instructions of the reverse transcription kit (AccurateBiology, AG11717), then the non‐specific reverse transcription of total RNA was completed. The total RNA extracted from HUVECs was carried out according to the reverse transcription kit (AccurateBiology, AG11707) instructions.

The ABI QuantiStudio7 Flex Realtime PCR system was used for RT‐PCR reaction with SYBR Green (Accurate Biology, AG11719) according to the standard procedure. Primers were designed by the online website of Sangon Biotech (https://www.sangon.com/). The list of primers is as follows:

**Table:** 

RNA		Primer sequence
miR‐155‐5p	RT	GTCGTATCCAGTGCAGGGTCCGAGGTATTCGCACTGGATACGACAACCCC
	F	ACCGAGGTTTAATGCTAATCGTG
	R	ATCCAGTGCAGGGTCCGAGG
miR‐515‐5p	RT	GTCGTATCCAGTGCAGGGTCCGAGGTATTCGCACTGGATACGACCAGAAA
	F	AACCTCCTTCTCCAAAAGAAAGC
	R	ATCCAGTGCAGGGTCCGAGG
miR‐135b‐5p	RT	GTCGTATCCAGTGCAGGGTCCGAGGTATTCGCACTGGATACGACTCACAT
	F	AACGGCTATGGCTTTTCATTCC
	R	ATCCAGTGCAGGGTCCGAGG
miR‐142–3p	RT	GTCGTATCCAGTGCAGGGTCCGAGGTATTCGCACTGGATACGACTCCATA
	F	AATCGGCGTGTAGTGTTTCCTA
	R	ATCCAGTGCAGGGTCCGAGG
miR‐26b‐3p	RT	GTCGTATCCAGTGCAGGGTCCGAGGTATTCGCACTGGATACGACAGCCAA
	F	AGGCGCCCTGTTCTCCATT
	R	ATCCAGTGCAGGGTCCGAGG
miR‐30a‐3p	RT	GTCGTATCCAGTGCAGGGTCCGAGGTATTCGCACTGGATACGACGCTGCA
	F	AACCGGCTTTCAGTCGGATG
	R	ATCCAGTGCAGGGTCCGAGG
U6	RT	GTCGTATCCAGTGCAGGGTCCGAGGTATTCGCACTGGATACGACAAAATA
	F	AGAGAAGATTAGCATGGCCCCTG
	R	CAGTGCAGGGTCCGAGGT
HIF‐1α	F	CCATTAGAAAGCAGTTCCGCAAGC
	R	GTGGTAGTGGTGGCATTAGCAGTAG
VEGFA	F	GCCTTGCCTTGCTGCTCTACC
	R	CTTCGTGATGATTCTGCCCTCCTC
β‐tubulin	F	ACGGTGGTGGAGCCCTACAAC
	R	GCGGAAGCAGATGTCGTAGAGC

### 2.6 High‐Throughput Sequencing and Bioinformatics Analysis

The quality of the cDNA library was determined by Agilent 2100 Bioanalyzer and the mixed sequencing library was denatured by 0.1 m NaOH to generate single‐strand DNA, then captured on Illumina flow cell and amplified into cluster in situ. According to the manufacturer's instructions, 51 cycles were run on Illumina NextSeq 500 sequencer.

After sequencing was completed, Solexa CHASTITY was used for quality control (QC), the original reads were screened to get clean reads, and whether the sequencing data could be used for subsequent data analysis was evaluated. The clean reads were disjointed by cutadapt (removing the 3′ splice sequence and short fragment), and the tag with length > = 15 nt was left to get the trimmed reads. The miRDeep2 software analysed all trimmed reads (compared with the reference genome) to quantify known miRNA and predict new miRNA, and the counts per million (CPM) value representing miRNA quantification can be obtained. Then, we used edgeR software to calculate the CPM value differentiation, screen the differential miRNAs, and draw the miRNA cluster diagram. The target genes of differential miRNA were counted by the miRNA target gene database, and Gene Ontology (GO) and Kyoto Encyclopedia of Genes and Genomes (KEGG) Pathway were analysed. Finally, a volcano plot and cluster plot were drawn to facilitate the data browsing. The miRNA sequencing data analysis procedure is shown in Figure S2.

### 2.7 Prediction and Identification of Target Genes for miR‐155‐5p

Target genes for miR‐155‐5p were predicted using the TargetScan. The identification was implemented by adopting the Dual‐Luciferase Reporter Assay. The putative target gene 3′‐UTR sequences including the miR‐155‐5p targeting site were inserted into the psiCHECK‐2 vector (HonorGene). Next, miR‐155‐5p mimic/mimic control and recombinant plasmid psiCHECK‐2‐3′UTR were co‐transfected into 293 T cells. Cells were cleaved after 36 h for dual luciferase reporter assay. The dual luciferase reporter assay was performed using Double‐Luciferase Reporter Assay Kit (Promega, E1910) in accordance with the manufacturer's instruction.

### 2.8 Cell Preparation and In Vitro Treatment

The HUVECs and AML‐12 cell lines used in this study were purchased from Procell Life Science & Technology Co. Ltd. HUVECs were cultured in Dulbecco's Modified Eagle Medium/Nutrient Mixture F‐12 (DMEM/F12, Gibco) containing 10% fetal bovine serum (FBS, Gibco), and AML‐12 cells were cultured in exosome‐free DMEM/F12 containing 10% FBS, 10 μg/mL insulin, 5.5 μg/mL transferrin, 5 ng/mL selenium and 40 ng/mL dexamethasone. The cells with stable growth states were treated under the culture conditions of 37°C, 95% humidity, and 5% CO_2_. After AML‐12 cells were treated with alcohol for 24 h, the AML‐Exos in the culture supernatant were extracted. AI‐Exos and AML‐Exos were added to HUVECs, and the concentration of exosome was 100 μg/mL. After 24 h of intervention, the cellular protein or RNA was extracted immediately. A wound healing assay was conducted by creating a scratch in a confluent cell monolayer with a sterile pipette tip. Cells were incubated in exosomes‐enriched medium, and images were taken at 0, 8, and 24 h. The migration rate was measured by the reduction in wound area using ImageJ software.

### 2.9 Statistical Analysis

All tests were conducted three times at least. The results were expressed as the mean ± standard deviation or SEM. SPSS and GraphPad Prism 8.0 were used for statistical analysis. All data were tested for normality and uniformity of variance; *t*‐test was used for comparison between the two groups; Fisher exact test and *χ* ^2^ test were used for GO classification and KEGG pathway prediction; one‐way analysis of variance (ANOVA) was used for comparison among groups; post hoc test was used for mean comparison between the two groups. *p* < 0.05 was considered statistically significant.

## 3 Results

### 3.1 Characterisation of AI‐Exos

**FIGURE 1:**
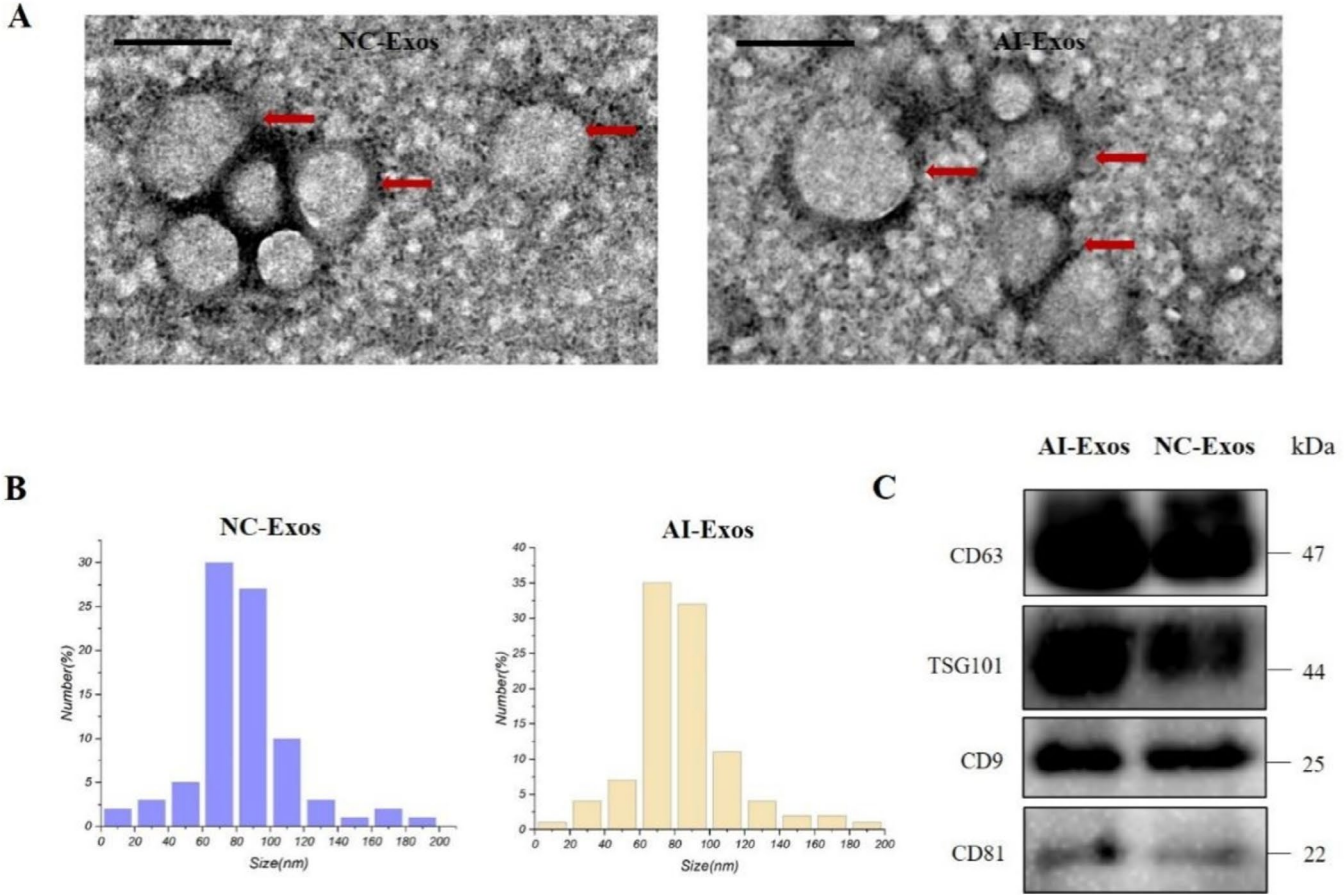
Characterisation of AI‐Exos. (A) Transmission electron microscopy (TEM) of NC‐Exos and AI‐Exos; scale: 100 nm. (B) Particle size distribution of NC‐Exos and AI‐Exos. (C) Western Blot identification of AI‐Exos and NC‐Exos.

The freshly extracted exosomes were characterised by TEM, NTA, and Western Blot. Morphologically, AI‐Exos appeared as cup‐shaped spherical vesicles in TEM images (Figure 1A), which were not significantly different from NC‐Exos. NTA analysis suggested that the size of AI‐Exos and NC‐Exos exhibited similar peaks around 80 nm (Figure 1B). The specific markers of exosomes, CD63, CD9, TSG101, and HSP90B1 (Figure 1C), were detected by Western Blot, with no significant differences observed between AI‐Exos and NC‐Exos. These results indicate that the exosomes were successfully extracted from human peripheral plasma.

### 3.2 The miRNA Profile of AI‐Exos Differentiated From NC‐Exos

**FIGURE 2:**
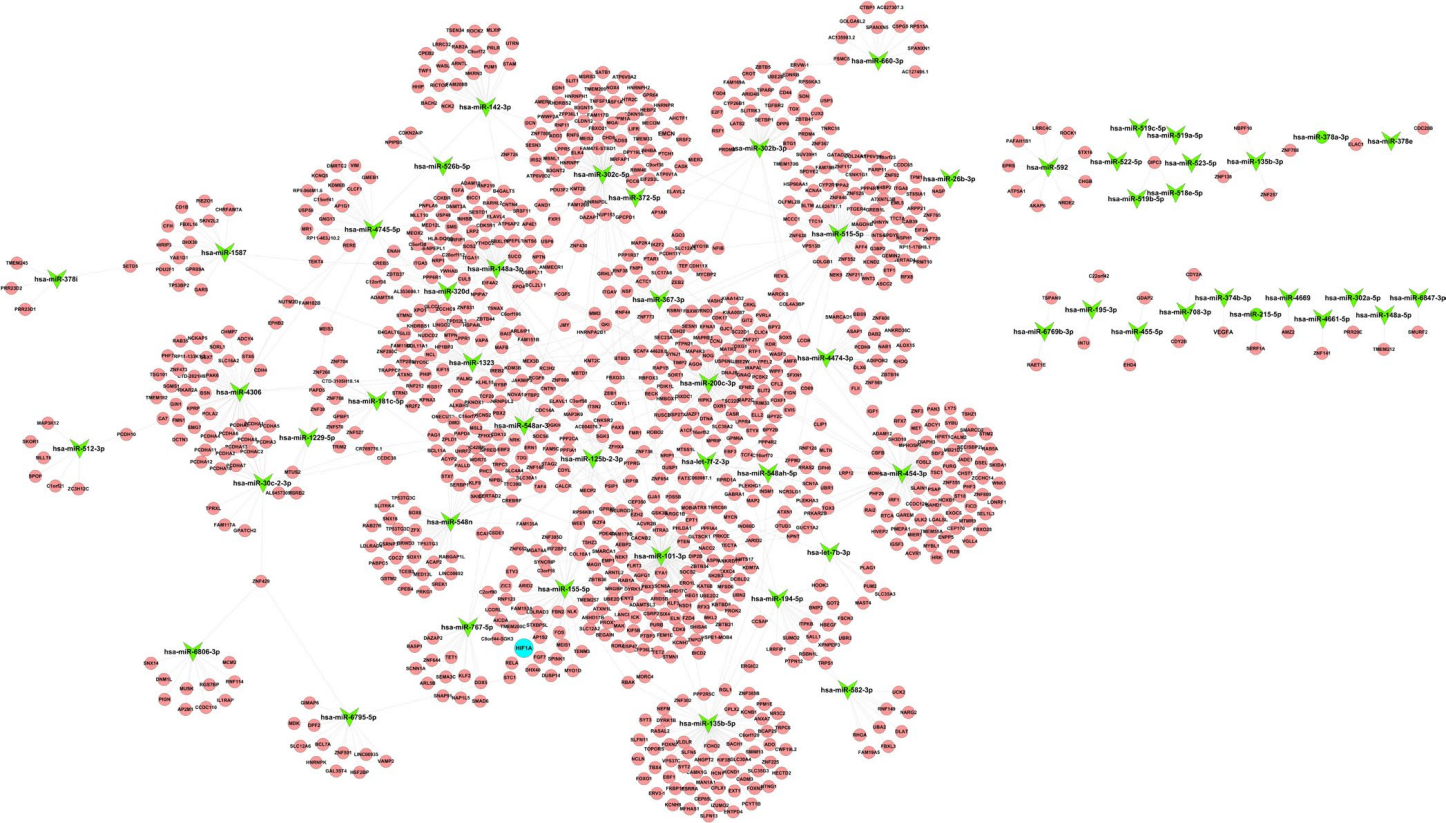
Networks of enriched pathways associated with 82 differentially expressed miRNAs.

**FIGURE 3:**
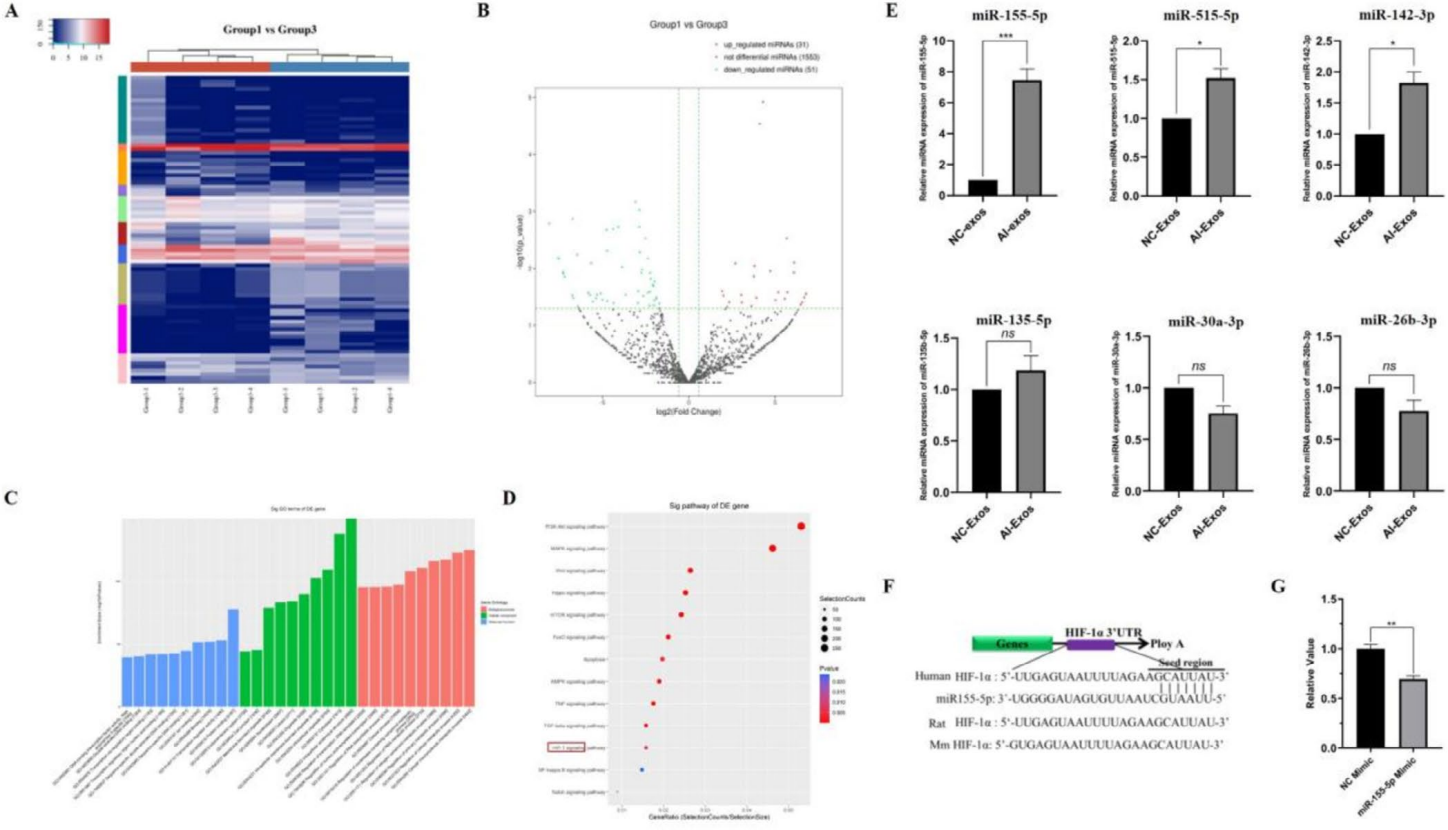
The miRNA profile of AI‐Exos differentiated from NC‐Exos. (A) Differentially expressed miRNAs cluster plot, each column represents a sample, each row represents a miRNA, and blue and red represent down‐regulated and up‐regulated miRNAs, respectively. (B) Differentially expressed miRNAs volcano plot, the horizontal axis represents the log2FoldChange value, the vertical axis represents −log10p value, and green and red dot represents down‐regulated and up‐regulated miRNAs, respectively. (C) Top 10 items in the results of GO analysis (BP, CC, MF), arranged from low to high according to the *p*‐value, and the ordinate represents the *p*‐value (−log10 conversion). (D) Top 13 items in the results of KEGG pathway analysis, the dot area represents miRNAs target genes number involved in the corresponding pathway, the abscissa indicates the proportion of the miRNAs target genes involved in the corresponding pathway, red indicates lower p‐value, blue indicates higher p‐value. (E) RT‐PCR verified the expression of miR‐155‐5p, miR‐515‐5p, miR‐142–3p, miR‐135b‐5p, miR‐30a‐3p and miR‐26b‐3p, respectively. ***means *p* < 0.001, * means *p* < 0.05, ns, no statistical difference; (F) TargetScan suggests that HIF‐1α is the target gene of miR‐155‐5p; (G) Dual‐Luciferase reporter assay showed the ratio of HIF‐1α were reduced when compared with the control group, suggested HIF‐1α might be the target gene of miR‐155‐5p in vivo.

Exosomes transport various signalling molecules for intercellular communication, with miRNA identified as a crucial player. To gain further insights into the miRNA profile of AI‐Exos, we performed miRNA high‐throughput sequencing. The results revealed that 82 miRNAs were differentially expressed in AI‐Exos, and the target gene network for all differentially expressed miRNAs was mapped (Figure 2). Specifically, 31 miRNAs were up‐regulated and 51 miRNAs were down‐regulated (|log_2_Foldchange| > 2, *p* < 0.01). A heat plot (Figure 3A) and a volcano plot (Figure 3B) of the differentially expressed miRNAs were generated. GO functional enrichment analysis includes three sub‐items: molecular function (MF), cellular component (CC), and biological process (BP). For MF, these differentially expressed miRNA functions are involved in protein binding, ion binding, and transcriptional activity regulation. For CC, cytoplasm and organelles were described as the most common. For BP, it mainly focused on the regulation of cell metabolism and transcription (Figure 3C). KEGG pathway enrichment analysis showed that 82 differentially expressed miRNAs were enriched in 151 pathways, including classical signalling pathways that regulate bone homeostasis such as PI3K, MAPK, Wnt, Hippo, mTOR, TGF‐β, HIF‐1 (Figure 3D).

Subsequently, some differentially expressed miRNAs were selected for RT‐PCR verification, including four up‐regulated miRNAs and two down‐regulated miRNAs. The results showed that miR‐155‐5p, miR‐515‐5p, miR‐142‐3p were consistent with the sequencing results, of which miR‐155‐5p was the most statistically significant, but there was no significant difference in miR‐135b‐5p, miR‐30a‐3p and miR‐26b‐3p (Figure 3E). Additionally, the gene sequence of HIF‐1α was highly coincident with miR‐155‐5p predicted by TargetScan, suggesting that miR‐155‐5p may silence the expression of HIF‐1α (Figure 3F). The relative luciferase activity (renilla luciferase/firely luciferase) of HIF‐1α was significantly reduced when compared with the control group (Figure 3G), indicating HIF‐1α were regulated by miR‐155‐5p in vivo.

### 3.3 MiR‐155‐5p Expression Up‐Regulated in AML‐Exos

**FIGURE 4:**
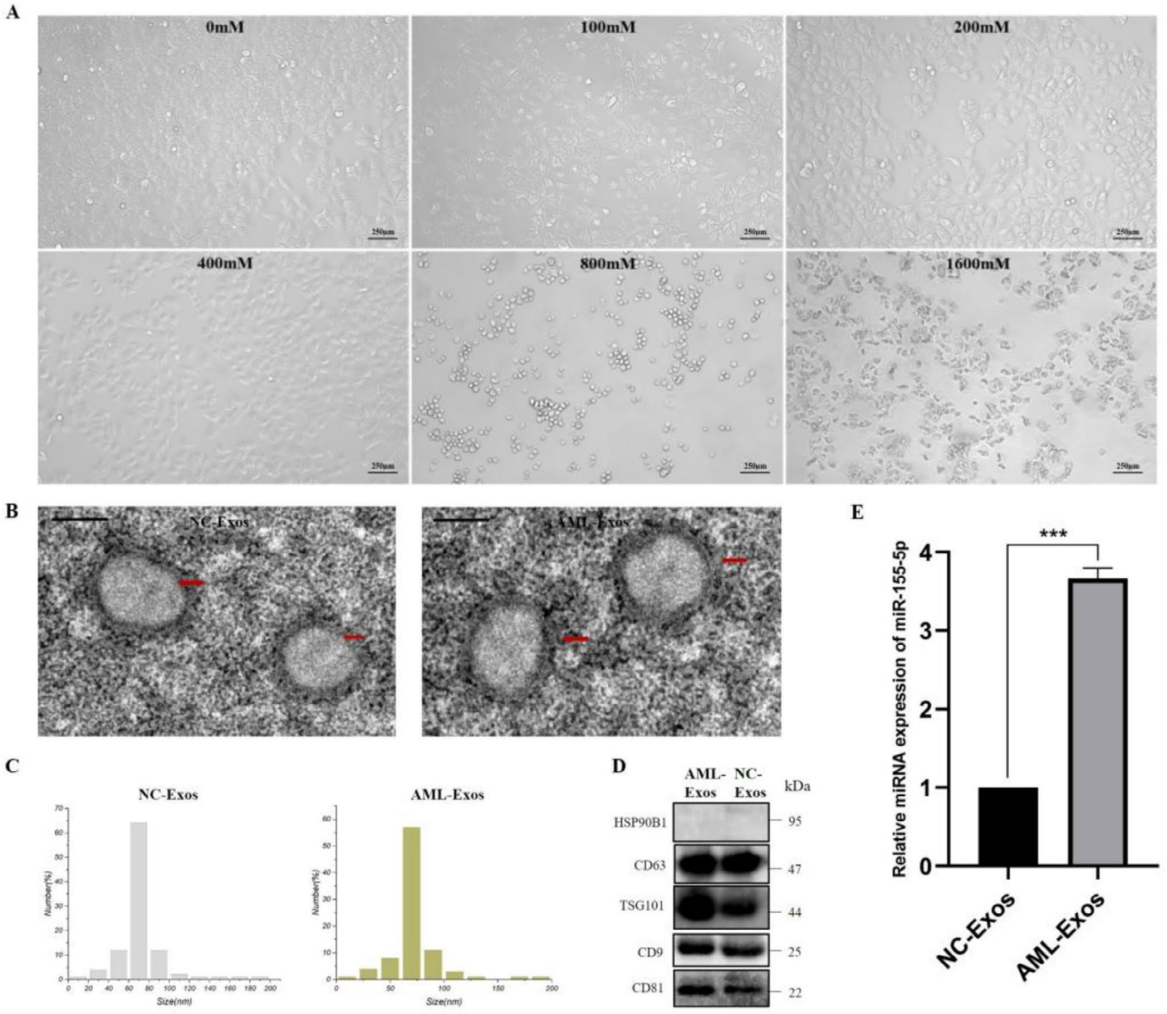
MiR‐155‐5p expression up‐regulated in AML‐Exos. (A) Morphology of AML‐12 cells under the intervention of different alcohol concentrations. (B–D) Characterisation of AML‐Exos. (E) RT‐PCR results showed that miR‐155‐5p was significantly up‐regulated in AML‐Exos compared with the exosomes secreted by normal AML‐12 cells (NC‐Exos).

AML‐12 hepatocytes were treated with alcohol at concentration of 0, 100, 200, 400, 800, and 1600 mM. The morphology of AML‐12 cells was observed under the microscope (Figure 4A). The maximum alcohol concentration (400 mM) that maintain the normal morphology of AML‐12 was selected for subsequent extraction of exosomes. Then freshly extracted exosomes were characterised by TEM, NTA, and Western Blot (Figure 4B–D). These results indicate that the exosomes were successfully extracted from the AML‐12 supernatant. The expression of miR‐155‐5p in AML‐Exos was detected by qRT‐PCR, and the results showed that miR‐155‐5p was significantly up‐regulated (Figure 4E).

### 3.4 AI‐Exos Inhibited the Proliferation and Migration and Promoted Apoptosis of HUVECs, Down‐Regulated HIF‐1α/VEGFA Expression

**FIGURE 5:**
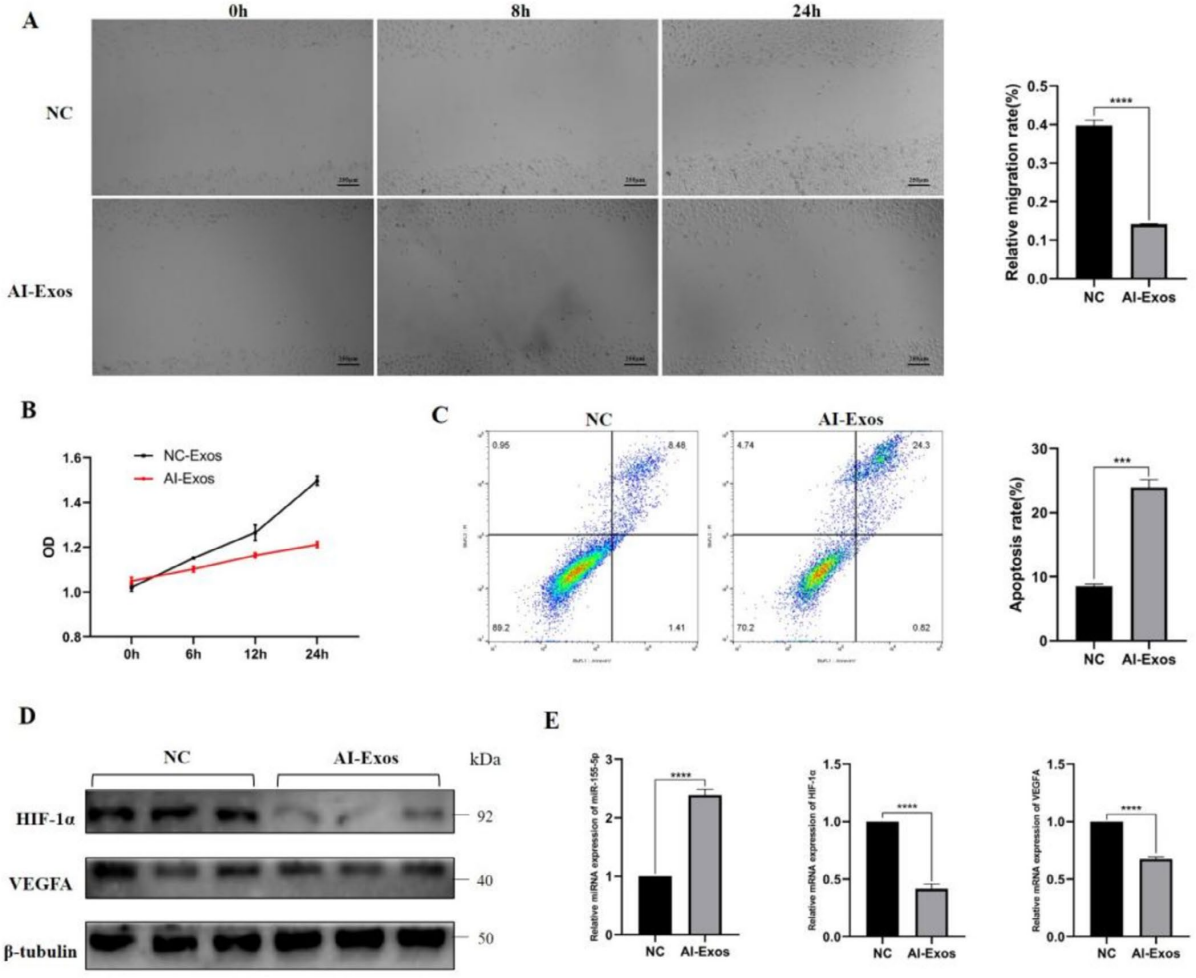
Effects of AI‐Exos on HUVECs. (A) The migration ability of HUVECs and its quantitative analysis was detected by wound healing assay. (B) The proliferation ability of HUVECs was detected by CCK‐8 assay. (C) The apoptosis of HUVECs and its quantitative analysis were detected by flow cytometry. (D) The expressions of HIF‐1α and VEGFA in HUVECs were detected by Western Blot. (E) The expression of miR‐155‐5p, HIF‐1α and VEGFA were detected by RT‐PCR.

The effect of AI‐Exos on the migration ability of HUVECs was detected by wound healing assay. The results showed that AI‐Exos significantly inhibited the migration ability of HUVECs at 24 h compared with NC‐Exos (Figure 5A). The proliferation ability of HUVECs detected by CCK‐8 assay showed that AI‐Exos inhibited the proliferation of HUVECs at all time points compared with NC‐Exos (Figure 5B). The apoptosis of HUVECs was detected by flow cytometry after Annexin V‐FITC/PI staining. The results showed that AI‐Exos significantly promoted apoptosis (Figure 5C). The results of Western Blot (Figure 5D) and RT‐PCR (Figure 5E) suggested that the expression of HIF‐1α/VEGFA decreased in HUVECs treated with AI‐Exos, with the expression of miR‐155‐5p significantly increased.

### 3.5 AML‐Exos inhibited the proliferation and migration and promoted apoptosis of HUVECs, down‐regulated HIF‐1α/VEGFA expression

**FIGURE 6:**
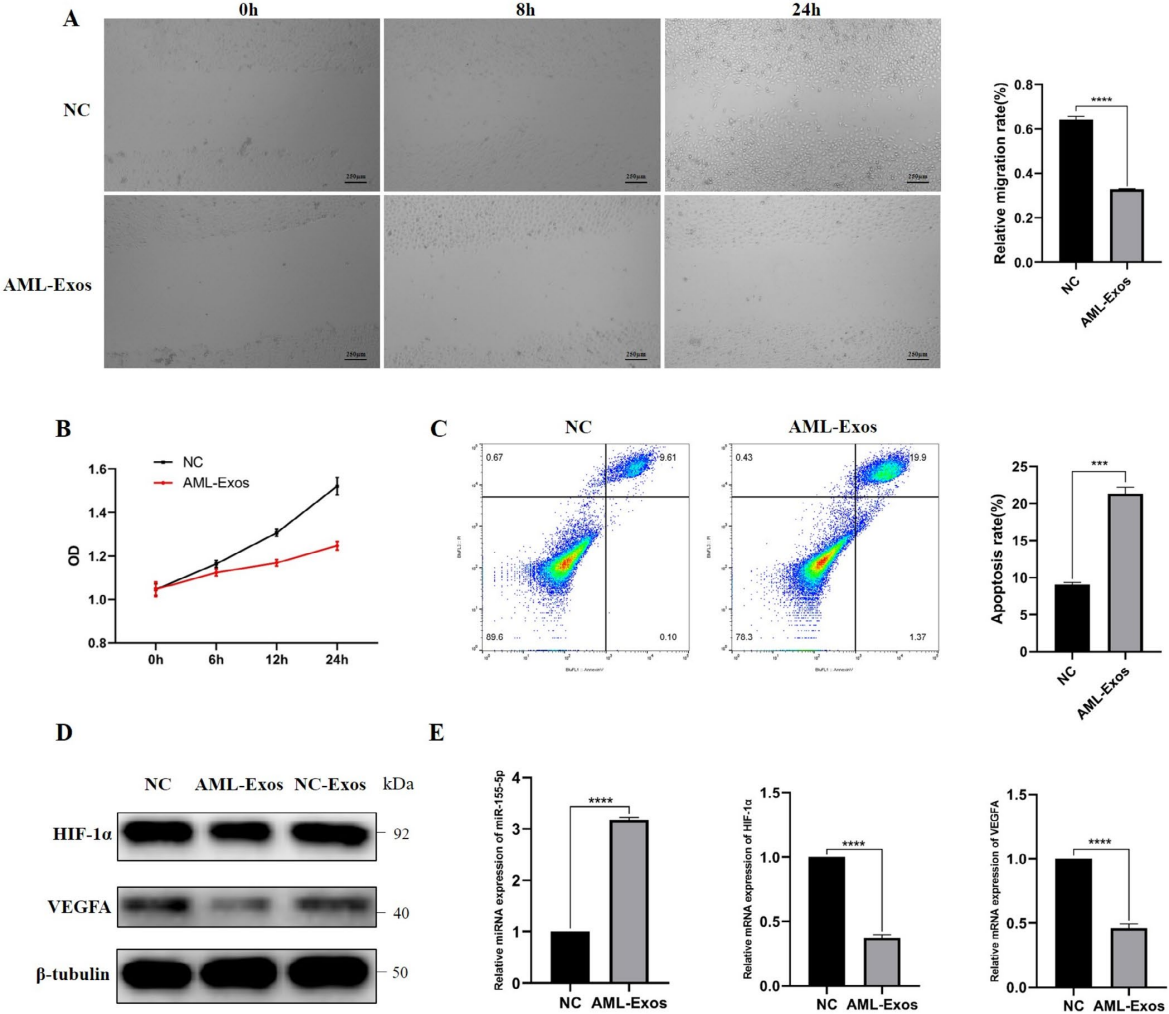
Effects of AML‐Exos on HUVECs. (A) The migration ability of HUVECs and its quantitative analysis was detected by wound healing assay. (B) The proliferation ability of HUVECs was detected by CCK‐8 assay. (C) The apoptosis of HUVECs and its quantitative analysis were detected by flow cytometry. (D) The expressions of HIF‐1α and VEGFA in HUVECs were detected by Western Blot. (E) The expression of miR‐155‐5p, HIF‐1α and VEGFA were detected by RT‐PCR.

The effect of AML‐Exos on the migration ability of HUVECs was detected by wound healing assay. The results showed that AML‐Exos significantly inhibited the migration ability of HUVECs at 24h compared with NC‐Exos (Figure 6A). The proliferation ability of HUVECs detected by CCK‐8 assay, indicated that AML‐Exos inhibited the proliferation of HUVECs at all time points compared with NC‐Exos (Figure 6B). The apoptosis of HUVECs was detected by flow cytometry after Annexin V‐FITC/PI staining. The results showed that AML‐Exos significantly promoted apoptosis (Figure 6C). Additionally, Western Blot (Figure 6D) and RT‐PCR (Figure 6E) results suggested that the expression of HIF‐1α and VEGFA decreased in HUVECs treated with AML‐Exos, while the expression of miR‐155‐5p significantly increased.

## 4 Discussion

As inter‐cellular messengers, exosomes not only contain the three main RNAs involved in protein synthesis but also carry abundant ncRNA, which plays important roles in the regulation of gene expression at both the transcriptional and post‐transcriptional levels [23]. Exosomes derived from human urine stem cells inhibit SONFH by transporting and releasing pro‐angiogenic gene DMBT1 and anti‐apoptotic gene TIMP1 [24]. Using high‐throughput sequencing, the role of miRNA carried by exosomes in regulating apoptosis, cell adhesion, and tumour angiogenesis has also been revealed in serum or tissue samples of osteosarcoma patients [25, 26]. The application of high‐throughput sequencing in AIONFH is mainly manifested in revealing gene expression profiles, developing new diagnostic markers, and predicting staging and prognosis [7, 10].

To better understand the pathogenesis of AIONFH and elucidate the gene expression characteristics and functional pathways, we studied the differential miRNAs in AI‐Exos by high‐throughput sequencing. The results showed that 82 miRNAs were differentially expressed in AI‐Exos, with 31 miRNAs up‐regulated and 51 miRNAs down‐regulated. Furthermore, we verified 6 specific miRNAs by RT‐PCR, with miR‐155‐5p showing the most statistically significant difference, suggesting that miR‐155‐5p may play an important role in the gene regulation of AIONFH. GO and KEGG analysis indicated these differentially expressed miRNAs were mainly involved in regulating bone homeostasis and angiogenesis. These specific miRNAs primarily function at the post‐transcriptional level, inhibiting their target genes by forming silent complexes, thus participating in the regulation of angiogenesis and osteogenesis in AIONFH. Additionally, TargetScan prediction suggested that HIF‐1α may be regulated by miR‐155‐5p, and the target relationship between miR‐155‐5p and HIF‐1α was validated via a dual‐luciferase assay.

Previous study has shown that under 1% O_2_ condition, the proliferation ability and colony formation efficiency of BMSCs decreased, while the osteogenic and chondrogenic differentiation ability increased [27]. Gene chip analysis of mRNA and miRNA expression in BMSCs under hypoxia revealed that HIF‐1α was significantly down‐regulated while miR‐155‐5p was significantly up‐regulated [27]. In addition, the expression of miR‐155 in the intestinal tissue of mice was up‐regulated under hypoxia [28]. Acute hypoxia inhibits prolyl hydroxylase (PHD) and stabilises HIF‐1α, but long‐term chronic hypoxia induces HIF‐1α “desensitisation”, indicating that HIF‐1α only temporarily up‐regulated under hypoxia [29, 30]. Such phenomenon may partly explain the inconsistency between BMSCs osteogenic differentiation and HIF‐1α expression in the above studies. Although the potential mechanism has not been fully elucidated, Ulrike et al. [28] have shown that the application of miRNA‐155 inhibitors helps to maintain the stability and activity of HIF‐1α in long‐term hypoxia. These results suggest that miR‐155‐5p can specifically silence HIF‐1α, resulting in cell dysfunction or disease. Additionally, the target relationship between miR‐155‐5p and HIF‐1α was validated via a dual‐luciferase assay, and our further study found that AI‐Exos down‐regulated the expression of HIF‐1α/VEGFA in VECs, accompanied by the up‐regulation of miR‐155‐5p in AI‐Exos and VECs, suggesting that AI‐Exos may affect the function of VECs by carrying miR‐155‐5p.

Previous studies have shown that the accumulated metabolites of alcohol, such as acetaldehyde and lipid peroxidation, not only damage the liver but also harm the endangium, resulting in arteriolar degeneration and hardening. This reduces the blood supply to the femoral head, causing osteocyte ischemia [4, 31]. At the same time, the direct cytotoxicity of alcohol and its metabolites may further damage ischemic bone cells, cause irreversible cell death, and eventually lead to ONFH [31]. Although most existing studies emphasise that the systemic effect of alcohol on osteonecrosis may be closely related to liver metabolism, the exact pathogenesis of AIONFH is still unclear [31, 32]. However, the positive correlation between the incidence of AIONFH and the genetic variation of alcohol dehydrogenase (ADH) and acetaldehyde dehydrogenase (ALDH) in peripheral blood and organs in the East Asian population seems to support this view [20, 21]. These results suggest that the effects of alcohol on the liver and blood vessels may be involved in AIONFH occurrence.

In this study, we found that the expression of miR‐155‐5p in AML‐Exos increased specifically under the alcohol‐stimulated condition, consistent with the results observed in AI‐Exos, suggesting that AML‐Exos may be involved in the formation of AI‐Exos. Furthermore, AML‐Exos inhibited the migration and proliferation of VECs, promoted its apoptosis, and down‐regulated the expression of HIF‐1α/VEGFA via up‐regulated the expression of miR‐155‐5p, which was consistent with the effect of AI‐Exos on VECs. These inspiring results provide a theoretical basis for a scientific hypothesis that exosomes released from alcohol‐stimulated hepatocytes affect the function of VECs by carrying miR‐155‐5p, laying a foundation for further exploration of the pathogenesis of AIONFH.

**FIGURE 7:**
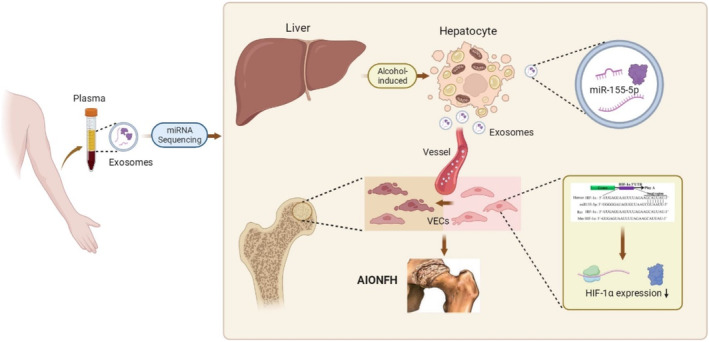
The proposed possible pathogenesis of AIONFH based on our study.

Inevitably, there are some shortcomings in our study. It is unknown how miR‐155‐5p carried by exosomes released from alcohol‐stimulated hepatocytes specifically transports to the microvessels of the femoral head and inhibits the HIF‐1α/VEGFA pathway, thus participating in AIONFH occurrence. Further animal experiments are needed to explore the specific role of exosomes released from alcohol‐stimulated hepatocytes in the local part of the femoral head, which is also the direction that we will further study in the future (Figure 7).

## 5 Conclusions

In conclusion, our study is the first to reveal the expression profile of miRNAs in plasma exosomes of AIONFH patients and propose a possible mechanism by which exosomes released from alcohol‐stimulated hepatocytes regulate VECs in AIONFH.

## Author Contributions

**Wei Chen:** data curation (lead), methodology (lead), writing – original draft (lead). **Zhe Yi:** validation (supporting), writing – original draft (supporting). **Gaojie Luo:** data curation (supporting), methodology (supporting). **Peiyao Zhang:** methodology (supporting), visualization (supporting). **Panfeng Wu:** funding acquisition (supporting), resources (supporting), supervision (supporting). **Fang Yu:** supervision (supporting). **Liming Qing:** conceptualization (lead), funding acquisition (supporting), project administration (equal), supervision (equal), visualization (supporting), writing – review and editing (lead). **Juyu Tang:** funding acquisition (lead), project administration (lead), resources (lead), supervision (lead), writing – review and editing (supporting).

## Ethics Statement

This study was approved by the Ethical Committee of Xiangya Hospital Central South University (no.202004217).

## Conflicts of Interest

The authors declare no conflicts of interest.

## Supporting information


**Data S1.** Supporting Information.

## Data Availability

The data that support the findings of this study are available from the corresponding author upon reasonable request.
