# Supplementary material for: MicroRNA Expression Profile in the Patient's Plasma Exosomes of Alcohol‐Induced Osteonecrosis of Femoral Head: Potential Vascular Regulation Mechanism
Source: J Cell Mol Med. 2025 Feb 24;29(4):e70382. doi: 10.1111/jcmm.70382 (PMC11850087; doi:10.1111/jcmm.70382)
Supplement: Supplementary file 1 — Data S1. Supporting Information. [file JCMM-29-e70382-s001.docx]

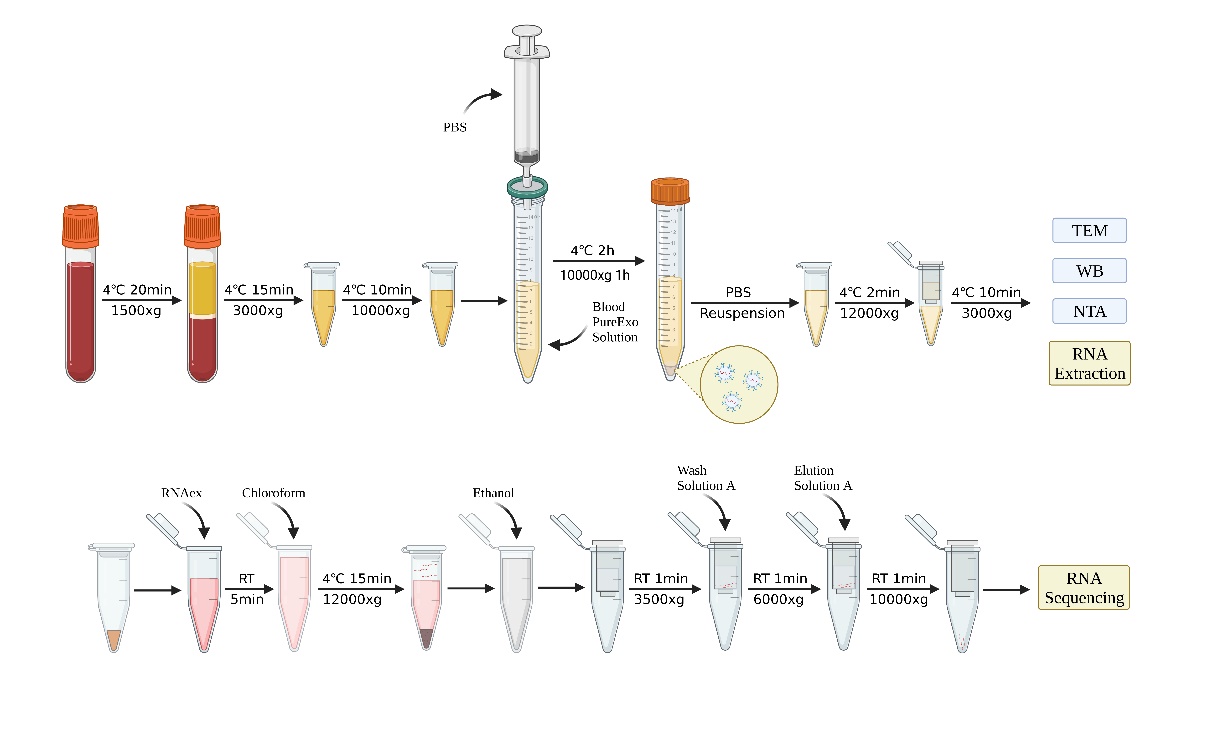


**Fig. S1** Human plasma exosomes and total RNA extraction process.


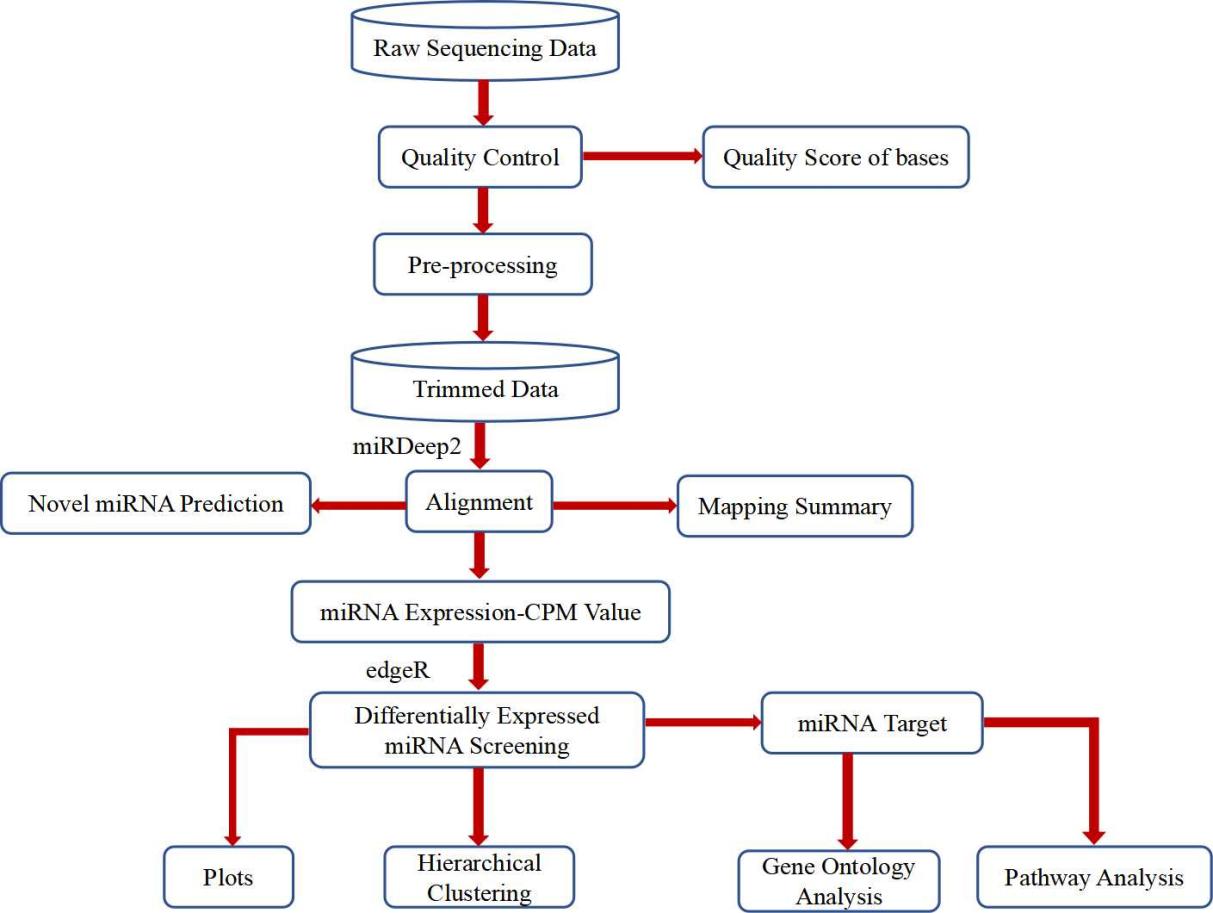


**Fig. S2** MiRNA sequencing data analysis procedure.
